# Can ChatGPT Help General Practitioners Become Acquainted with Conversations About Dying? A Simulated Single-Case Study

**DOI:** 10.3390/healthcare13070835

**Published:** 2025-04-06

**Authors:** Filipe Prazeres

**Affiliations:** 1 Faculty of Health Sciences, University of Beira Interior, 6200-506 Covilhã, Portugal; filipeprazeresmd@gmail.com; 2 Family Health Unit Beira Ria, 3830-596 Gafanha da Nazaré, Portugal; 3 RISE-Health, Department of Community Medicine, Information and Health Decision Sciences, Faculty of Medicine, University of Porto, 4200-450 Porto, Portugal

**Keywords:** communication, death, general practitioners, artificial intelligence, ChatGPT-4o, palliative medicine

## Abstract

**Background/Objectives**: General practitioners (GPs) should be able to initiate open conversations about death with their patients. It is hypothesized that a change in attitude regarding discussions of death with patients may be accomplished through doctors’ training, particularly with the use of artificial intelligence (AI). This study aimed to evaluate whether OpenAI’s ChatGPT can simulate a medical communication scenario involving a GP consulting a patient who is dying at home. **Methods**: ChatGPT-4o was prompted to generate a medical communication scenario in which a GP consults with a patient dying at home. ChatGPT-4o was instructed to follow seven predefined steps from an evidence-based model for discussing dying with patients and their family caregivers. The output was assessed by comparing each step of the conversation to the model’s recommendations. **Results**: ChatGPT-4o created a seven-step scenario based on the initial prompt and addressed almost all intended recommendations. However, two points were not addressed: ChatGPT-4o did not use terms like “dying”, “passing away”, or “death”, although the concept was present from the beginning of the conversation with the patient. Additionally, cultural and religious backgrounds related to dying and death were not discussed. **Conclusions**: ChatGPT-4o can be used as a supportive tool for introducing GPs to the language and sequencing of speech acts that form a successful foundation for meaningful, sensitive conversations about dying, without requiring advanced technical resources and without placing any burden on real patients.

## 1. Introduction

Nyatanga [1] wrote in 2012 that while general practitioners (GPs) may wish to discuss death with patients, time constraints often make it difficult, and since palliative care represents only a small part of their clinical care duties, they may lack the necessary knowledge and skills in this area. Thus, not all GPs initiate open conversations about death since some may feel unprepared [1]. Another reason GPs avoid telling terminally ill patients about their approaching death is that they see themselves primarily as healers, and this role conflicts with the harsh reality of the patient’s condition, which can feel hopeless [2]. Additionally, ethnic differences between doctors and patients have been identified as barriers to discussing end-of-life issues [3]. Since dying patients want physicians to be truthful regarding their disease and treatment options [4], developing the right knowledge and communication skills is vital to improve this aspect of care and ensure a dignified death [1]. Improved communication between patients and physicians is linked to better outcomes, including less physical distress at the end of life, fewer high-intensity interventions, a lower likelihood of dying in the ICU, earlier and more frequent hospice referrals, and reduced medical costs [5].

Virtual patients provide a controlled and risk-free setting for learning, enabling repeated practice that is often challenging to achieve in real clinical training [6]. Thanks to recent advances in artificial intelligence (AI) and language technology, virtual patients powered by large language models are now available for educational use (e.g., the SOPHIE—Standardized Online Patient for Health Interaction Education—platform [7]).

Since most GPs receive limited training in serious illness communication, a conversational AI chatbot assistant could offer a valuable, risk-free environment to help them develop and refine their communication skills [8]. It is hypothesized that a change in GPs’ attitudes about discussing death with patients may be accomplished through doctors’ training, in particular with AI.

Launched on 30 November 2022, OpenAI’s Chat Generative Pre-trained Transformer (ChatGPT) has provided everyone with easy access to generative AI. Since then, it has garnered significant research attention, with 8731 entries on PubMed as of March 2025, and this number is expected to continue rising. In medical education, AI and generative language models can surpass medical candidates in exam performance [9] and can enhance learning by providing realistic simulations, virtual patients, personalized feedback, and improved evaluation methods and by overcoming language barriers [10].

Previous research has shown that ChatGPT’s language model can have a positive role in palliative care communication training [11] and can successfully assist in role-playing scenarios for breaking bad news in emergency medicine [12]. However, its use in GP medical education for discussing end-of-life conversations with patients has not been studied before.

Therefore, this study aimed to evaluate whether OpenAI’s ChatGPT-4o can be used to simulate a medical communication scenario in which a GP consults a patient dying at home.

## 2. Materials and Methods

OpenAI’s ChatGPT-4o was prompted to generate the following medical scenario: You are a family physician visiting a patient you know very well at his home. You are there to discuss the patient’s approaching death due to pancreatic cancer with him and his family caregiver, who is his daughter.

ChatGPT-4o was also informed that this conversation must follow seven steps: (i) conversation opening, (ii) situation assessment, (iii) needs analysis, (iv) goal setting, (v) action plan, (vi) review and summary, and (vii) conversation closing. These predefined steps were derived from a recently published evidence-based model by Felber et al. [13] for discussing dying with patients and their family caregivers (FCs).

The output was assessed by directly comparing each step of the conversation to the recommendations in Felber et al.’s [13] model for discussing dying, which served as the quality assessment for the simulated medical communication scenario.

The seven-step recommendations for end-of-life conversations by Felber et al. [13] have been fully described elsewhere [13]. Briefly, they ensure compassionate and structured communication between healthcare providers, patients, and FCs. It begins with a conversation opening, where contact is established and key concerns are understood. Situation assessment follows, evaluating the patient’s condition and acknowledging the likelihood of death in the coming days. Next, a needs analysis identifies the patients’ and FCs’ concerns regarding dying and death. Goal setting involves collaborative decision-making on treatment and care, ideally aiming for a dignified death while considering both hopeful and worst-case scenarios. Based on these goals, an action plan is developed, detailing care priorities, contingency planning, and key contacts. The conversation then moves to review and summary, ensuring information is clearly communicated and feedback is gathered. Finally, conversation closing allows space for questions, ensuring a respectful and supportive conclusion [13].

## 3. Results

The output data from ChatGPT-4o, along with its corresponding analysis, is presented in Table 1. ChatGPT-4o created a seven-step scenario based on the initial prompt. The comparison of each step of the conversation to the recommendations in the Felber et al. [13] model for discussing dying shows that the conversational AI covered almost all intended recommendations. However, two points were not addressed: ChatGPT-4o did not use words like “dying”, “passing away”, or “death”, though the concept was present from the start of the conversation with the patient, and cultural and religious backgrounds related to dying and death were not discussed.

## 4. Discussion

This single-case study successfully demonstrated that OpenAI’s ChatGPT-4o can effectively simulate a medical communication scenario in which a GP consults with a patient dying at home, addressing the needs of both the patient and his family caregiver by using a simple prompt and without the requirement of complex prompt engineering. Nonetheless, skilled prompt engineering may be useful in medical education [14], as it could facilitate more detailed conversations; however, this aspect was beyond the scope of the current study.

These findings are important because they demonstrate the potential of ChatGPT-4o as a supportive tool for introducing GPs to the language and sequencing of speech acts that form a successful foundation for meaningful, sensitive conversations about dying without requiring advanced technical means or prompt engineering knowledge. These results align with previous research conducted in the emergency setting that indicated that ChatGPT can be used to train physicians on breaking bad news to patients [12].

In end-of-life care, empathetic communication and responsiveness to the needs of both patients and caregivers are essential. ChatGPT has previously shown promising results by generating quality, empathetic responses to patient questions [15]. As a result, these AI tools can provide a safe place for GPs to practice and improve their end-of-life discussions without placing any burden on real patients.

This study had several limitations. First, only one interaction with ChatGPT was evaluated, and it is uncertain whether the success would be maintained with repeated interactions. Second, while following the seven steps from Felber et al. [9] helped guide ChatGPT in structuring the conversation, strict adherence may have missed opportunities to adapt fluidly to the scenario based on the patient’s needs. Third, ChatGPT was not instructed to consider cultural, religious, or personal beliefs about death and dying, which are highly individualized and may have influenced the final conversation output. Lastly, no comparison with different ChatGPT versions or other conversational AI chatbots was performed.

Nonetheless, this single-case study not only serves as an early-stage exploration before larger studies but also provides an in-depth analysis of a sensitive conversation. Additionally, it demonstrates the ease of creating simulated cases for GPs and aims to help them overcome the discomfort of speaking openly with their dying patients by gaining exposure to this topic and encouraging reflection.

In future studies, researchers could involve real patients and caregivers to provide feedback on simulated consultations, thereby evaluating the realism of AI interactions rather than simply comparing the responses to recommendations, as was performed in the present study. Additionally, it is important to examine whether the use of ChatGPT and other conversational AI chatbots in training correlates with measurable improvements in GPs’ communication skills over time in actual healthcare settings.

## 5. Conclusions

ChatGPT-4o can serve as a valuable tool to help GPs become acquainted with conversations about dying. It can provide structured support for role-playing sensitive medical discussions without requiring advanced technical resources or placing any burden on real patients. While ChatGPT-4o can assist in simulating these scenarios, it cannot replace human interaction, as only a GP familiar with the patient’s background can effectively integrate emotional cues, cultural sensitivities, and ethical responsibility into his/her interactions.

## Figures and Tables

**Table 1:** The seven steps of the conversation by ChatGPT-4o and their corresponding analysis.

Steps of the Conversation	Analysis of ChatGPT’s Output
Conversation Opening	
(As you enter the room, you offer a gentle smile, and approach the patient’s bed.) Doctor: Hello, Mr. Jones. It’s good to see you. (You extend a warm hand to his daughter.) And you too, Sarah. How are you both doing today? (You pause, giving them space to express their feelings.) Sarah (daughter): We’re managing… but it’s been hard. Dad’s been more tired than usual, and he’s having more pain. I just… I don’t know what to expect. Mr. Jones (patient): I know what’s coming, doc. But I’m not ready. I don’t want to be in pain. Doctor: I hear you both. This is a difficult time, and it’s completely natural to feel uncertain and scared. Let’s talk through it together and make sure you both feel supported and that Mr. Jones is comfortable.	The doctor addressed both the patient and the daughter. The doctor was consciously present during the conversation. The doctor kept the conversation short and simple. No time limits were predefined for the consultation. As this was a home visit, it made sense not to impose time constraints, as the doctor’s presence would have been arranged in advance.
2. Situation Assessment	
Doctor: Mr. Jones, I know you’ve been through a lot with the treatments and the ups and downs. From my perspective, I think we are nearing the final phase of this illness. I don’t expect it to be long now, possibly just days. How do you feel about where things are right now? Mr. Jones: I’ve had some good days and some bad, but I’ve noticed it’s been harder to breathe lately. I’m scared of what’s next. Am I going to… suffer? Doctor: I understand your fear, and I want to reassure you that we’re here to make sure you don’t suffer. My main goal is to keep you as comfortable as possible. We’ll continue managing your symptoms, especially the pain and any discomfort. (Turning to Sarah) Sarah, how are you feeling about this? Sarah: I’ve been trying to prepare for this, but… I don’t know how to let go. I don’t want him to be in pain, and I don’t know what to do when… when things get worse. Doctor: It’s okay to feel that way. This is an emotional time for both of you, and there’s no right way to go through it. We’ll work together to make sure you feel supported too.	The doctor began by asking the patient for his evaluation. The doctor addressed the patient’s approaching death. The doctor used an appropriate questioning style and answered questions honestly and empathetically. The doctor acknowledged the emotions expressed by both the patient and the daughter. Although the doctor did not use words like “dying”, “passing away”, or “death”, the notion was present from the start of the conversation with the patient.
3. Needs Analysis	
Doctor: Let’s talk about what’s most important for you right now, Mr. Jones. What are your biggest concerns as you think about the days ahead? Mr. Jones: I don’t want to be in pain, and I don’t want to be a burden to Sarah. I also want to stay at home if possible. Doctor: Those are all things we can focus on. We can work together to control the pain and make sure Sarah has the support she needs. (Turning to Sarah) Sarah, I know caring for your father has been overwhelming at times. What are your biggest concerns as we approach this stage? Sarah: I’m worried about not knowing what to do, like when to call for help or how to keep him comfortable. I’m afraid I’ll miss something important. Doctor: That’s understandable. We’ll make sure you know what to expect and when to reach out for help. We’ll also make sure you’re not alone in this.	The doctor focused on asking what was most important to the patient and his daughter and inquired about their needs regarding the dying phase.
4. Goal Setting	
Doctor: Mr. Jones, our main goal right now is to ensure you can pass peacefully and comfortably, with as little pain as possible. Would you agree that’s what we should aim for? Mr. Jones: Yes. That’s what I want. Doctor: And Sarah, it sounds like your goal is to make sure your father is comfortable and to feel prepared for what’s ahead. Is that right? Sarah: Yes, I just don’t want him to suffer. Doctor: Okay, so let’s talk about how we can work towards these goals. We’ll focus on keeping you comfortable, Mr. Jones, with medications that control pain, anxiety, and shortness of breath. Sarah, we’ll also make sure you have clear instructions and someone you can contact 24/7 in case anything changes.	The doctor clarified the goals for the patient’s final days. The doctor included the family caregiver in the conversation. The preferred place of death was not discussed in this part of the conversation, but the patient had previously informed the doctor of the desire to stay at home if possible. Cultural and religious backgrounds related to dying and death were not addressed.
5. Action Plan	
Doctor: So here’s the plan: we’ll adjust your medications, Mr. Jones, to make sure your pain is well controlled. If you experience any symptoms, like trouble breathing or anxiety, we’ll have medications ready for that too. (Turning to Sarah) Sarah, we’ll set up home hospice services if they’re not in place already. This means a nurse can come by regularly, and you’ll have access to help any time of the day or night. If Mr. Jones’ condition changes or if there’s anything you’re unsure about, you can call the hospice team. I’ll also be available to check in. Sarah: That helps, knowing someone will be here when we need it. Doctor: We’ll also prepare for the possibility that things may change quickly. If you’re hoping for the best but planning for the worst, we’ll have a backup plan. That way, you’ll know what to expect no matter what happens.	The doctor created a reliable care plan based on prioritized needs and involved the patient’ s daughter in the process.
6. Review and Summary	
Doctor: Just to review, our main focus now is on keeping you comfortable, Mr. Jones. Sarah, we’ll ensure you’re supported as well. I’ll update the hospice team on the changes in Mr. Jones’ condition, and they’ll visit regularly to help you manage. (Looking at both of them) Does this plan feel right for you? Is there anything we’ve missed? Mr. Jones: That sounds good, doc. I just want to rest now. Sarah: It helps to have a plan. Thank you for explaining everything so clearly. Doctor: I’m glad. You’re both doing incredibly well, considering the circumstances.	The doctor clarified the sharing of information with the hospice team in case the patient’s health deteriorated and reviewed the care plan with those present.
7. Conversation Closing	
Doctor: Before I go, do you have any more questions? Or anything else you’d like to talk about? Sarah: No… I think we’re okay for now. Just, thank you. Doctor: Of course. I’m here whenever you need me. (You gently place your hand on Sarah’s shoulder, then Mr. Jones’ hand.) You both have my full support, and I’ll check in again soon. (With a quiet nod and reassuring eye contact, you stand up to leave, letting the room settle into a calm and quiet space.)	The doctor allowed time for open questions and remained present and respectful when closing the conversation.

## Data Availability

The original contributions presented in this study are included in the article. Further inquiries can be directed to the corresponding author.

## References

[1] Nyatanga B. (2012). Are GPs ready to talk about death and dying?. Br. J. Community Nurs..

[2] Todd C.J., Still A.W. (1984). Communication between general practitioners and patients dying at home. Soc. Sci. Med..

[3] Periyakoil V.S., Neri E., Kraemer H. (2015). No Easy Talk: A Mixed Methods Study of Doctor Reported Barriers to Conducting Effective End-of-Life Conversations with Diverse Patients. PLoS ONE.

[4] Kuhl D., Stanbrook M.B., Hebert P.C. (2010). What people want at the end of life. CMAJ.

[5] Zhang B., Wright A.A., Huskamp H.A., Nilsson M.E., Maciejewski M.L., Earle C.C., Block S.D., Maciejewski P.K., Prigerson H.G. (2009). Health care costs in the last week of life: Associations with end-of-life conversations. Arch. Intern. Med..

[6] Byrne M., Cochrane T., Narayan V., Brown C., MacCallum K., Bone E., Deneen C., Vanderburg R., Hurren B. (2023). Empathic communication training in healthcare using Virtual Patients. People, Partnerships and Pedagogies.

[7] Haut K.G., Epstein R., Carroll T.M., Kane B., Schubert L., Hoque E. (2024). SOPHIE: Testing a Virtual, Interactive, AI-Augmented End-of-Life Communication Training Tool (RP122). J. Pain Symptom Manag..

[8] Phillips M., Vermylen J., Ballard H. (2024). From Bytes to Empathy: Can ChatGPT Teach Anesthesiologists How to Deliver Bad News?. Acad. Med. J. Assoc. Am. Med. Coll..

[9] Prazeres F. (2025). ChatGPT’s Performance on Portuguese Medical Examination Questions: Comparative Analysis of ChatGPT-3.5 Turbo and ChatGPT-4o Mini. JMIR Med. Educ..

[10] Karabacak M., Ozkara B.B., Margetis K., Wintermark M., Bisdas S. (2023). The Advent of Generative Language Models in Medical Education. JMIR Med. Educ..

[11] Srivastava R., Srivastava S. (2023). Can Artificial Intelligence aid communication? Considering the possibilities of GPT-3 in Palliative care. Indian J. Palliat. Care.

[12] Webb J.J. (2023). Proof of Concept: Using ChatGPT to Teach Emergency Physicians How to Break Bad News. Cureus.

[13] Felber S.J., Zambrano S.C., Guffi T., Schmitz F.M., Brem B.G., Schnabel K.P., Guttormsen S., Eychmuller S. (2024). How to talk about dying? The development of an evidence-based model for communication with patients in their last days of life and their family caregivers. PEC Innov..

[14] Heston T., Khun C. (2023). Prompt Engineering in Medical Education. Int. Med. Educ..

[15] Ayers J.W., Poliak A., Dredze M., Leas E.C., Zhu Z., Kelley J.B., Faix D.J., Goodman A.M., Longhurst C.A., Hogarth M. (2023). Comparing Physician and Artificial Intelligence Chatbot Responses to Patient Questions Posted to a Public Social Media Forum. JAMA Intern. Med..

